# Ortner's syndrome: - case series and literature review

**DOI:** 10.1590/S1808-86942011000500004

**Published:** 2015-10-22

**Authors:** Vijayalakshmi Subramaniam, Adarsha Herle TV, Navisha Mohammed, Muhammad Thahir

**Affiliations:** 1 MBBS, DLO, Dip NB (Assistant Professor); 2 MBBS, DLO, Dip NB (Consultant ENT Surgeon); 3 MBBS, DLO, Dip NB (Consultant ENT Surgeon ); 4 MBBS, MS (Professor)

**Keywords:** cardiovascular diseases, hoarseness, recurrent laryngeal nerve

## Abstract

**Abstract:**

More than a century ago, Ortner described a case of cardiovocal syndrome wherein he attributed a case of left vocal fold immobility to compression of the recurrent laryngeal nerve by a dilated left atrium in a patient with mitral valve stenosis. Since then, the term Ortner's syndrome has come to encompass any nonmalignant, cardiac, intrathoracic process that results in embarrassment of either recurrent laryngeal nerve-usually by stretching, pulling, or compression; and causes vocal fold paralysis. Not surprisingly, the left recurrent laryngeal nerve, with its longer course around the aortic arch, is more frequently involved than the right nerve, which passes around the subclavian artery.

**Objectives:**

To discuss the pathogenesis of hoarseness resulting from cardiovascular disorders involving the recurrent laryngeal nerve along with the findings of literature review.

**Materials and methods:**

This paper reports a series of four cases of Ortner's syndrome occurring due to different causes.

**Design:**

Case study.

**Result:**

Ortner's syndrome could be a cause of hoarseness of voice in patients with cardiovascular diseases.

**Conclusion:**

Although hoarseness of voice is frequently encountered in the Otolaryngology outpatient department, cardiovascular- related hoarseness is an unusual presentation. Indirect laryngoscopy should be routinely performed in all cases of heart disease.

## INTRODUCTION

Hoarseness of voice due to left recurrent laryngeal nerve paralysis was first described in 1897 by Norbert Ortner, an Austrian physician, in a patient with mitral valve disease (mitral stenosis and left atrial enlargement). Various cardiopulmonary conditions associated with left recurrent laryngeal nerve palsy, have been described, over the last 100 years. Thus, the syndrome is now also termed as cardiovocal syndrome.

Here a series of four different case reports of Ortner's syndrome occurring due to mitral stenosis, combined mitral stenosis with mitral regurgitation, corpulmonale with secondary pulmonary hypertension and aneurysm of the arch of aorta are presented. The findings of review of literature relating to cardiovascular disorders affecting the recurrent laryngeal nerve and the pathogenesis of hoarseness are also discussed.

## CASE 1

A 38-year old male presented with hoarseness of voice and cough with expectoration of 2 months duration. He also reported exertional dyspnoea for the last 2 years. He was never a smoker. Physical examination showed a normal pulse and blood pressure. The apex beat was tapping in character and was shifted down and out from the normal position. Clinical evidence of right ventricular hypertrophy was revealed through a left parasternal heave. The pulmonary component of the second heart sound was palpable. On auscultation, the first heart sound and the pulmonary component of the second heart sound were loud. A rumbling mid-diastolic murmur was heard in the apical area. Ear, Nose and Throat (ENT) examination revealed a paralysed left vocal cord in paramedian position. Chest X-ray Posteroanterior (PA) view showed cardiomegaly. Two Dimensional and M-mode (Timemotion mode) Echocardiography showed a dilated left atrium and pulmonary artery (32mm) with a calcified mitral valve (Mitral Valve Orifice Area = 0.634 cm^2^). The calculated pulmonary artery pressure was 48 mm of Hg. Mild aortic stenosis was also noted.

## CASE 2

A 17-year old boy diagnosed to have rheumatic heart disease (mitral stenosis with mitral regurgitation) was referred to the ENT clinic for hoarseness of voice of 7 months duration. On examination, he had a raised jugular venous pressure (JVP). Apex beat was localised in the left 5^th^ intercostal space. A pansystolic murmur of grade III/VI was heard in the mitral area radiating to the axilla and also a localised mid-diastolic murmur at the apex. Indirect laryngoscopy showed paralysed left vocal cord. Echocardiography showed a dilated left atrium with tight mitral stenosis and mitral regurgitation.

## CASE 3

**Figure 1:**
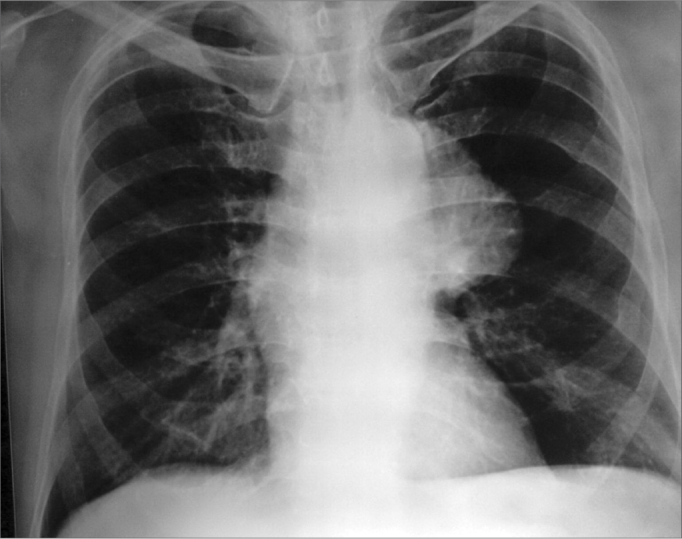
Chest X-ray PA view showing well- defined lobulated mediastinal lesion on the left side.

**Figure 2:**
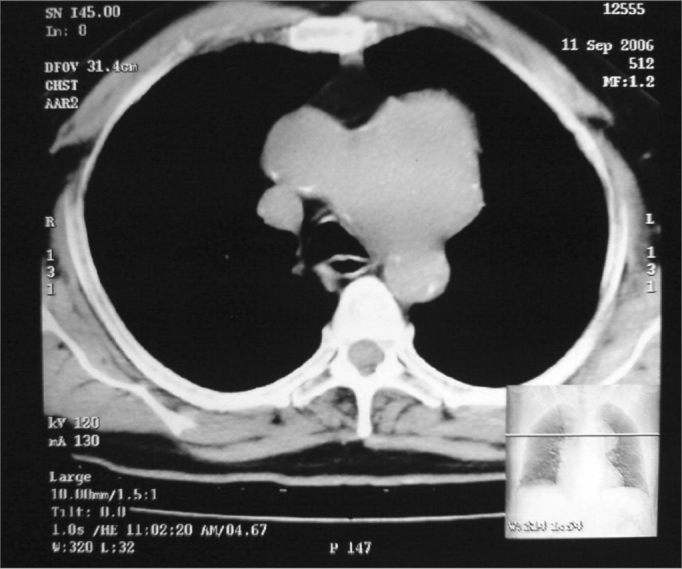
Plain CT scan chest, axial view showing large lobulated mass in the anterior mediastinum.

A 66-year old male, on treatment for hypertension for the last 20 years, presented to the ENT clinic complaining of hoarseness for over 8 months, apparently precipitated by an attack of common cold. He was a smoker for the last 40 years and did not have any other symptoms such as cough, dyspnoea, dysphagia or weight loss. On examination, a prominent pulsation was visible on the left side of the patient's neck. There was no cervical lymphadenopathy. Indirect laryngoscopy revealed a paralysed left vocal cord in paramedian position. Chest X-ray PA view showed a well-defined, lobulated mediastinal lesion on the left side (Figure 1). Contrast enhanced Computerised Tomography (CECT) image showed a large, lobulated, enhancing saccular aneurysm arising from the arch of aorta with peripheral thrombus (Figures 2 e 3).

**Figure 3:**
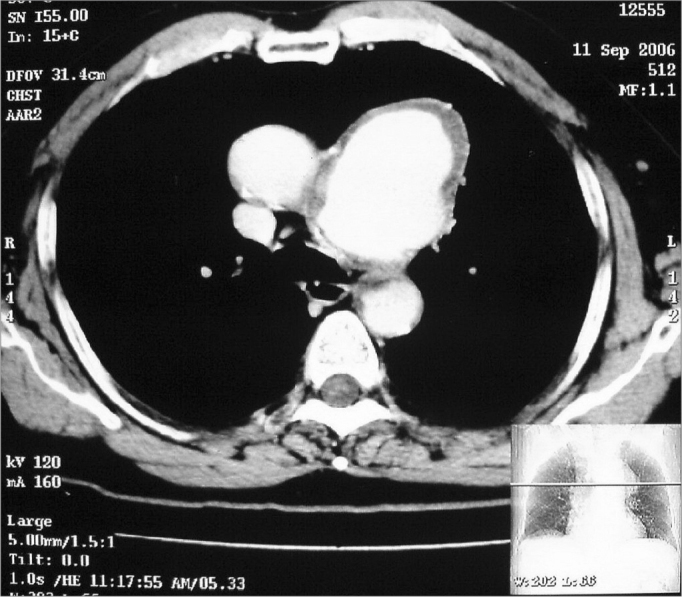
Contrast enhanced CT scan chest, axial view showing large, lobulated, enhancing saccular aneurysm arising from the arch of aorta with peripheral thrombus.

## CASE 4

A 55-year old male patient presented with hoarseness of voice of 8 months duration. He was a chronic smoker and previously diagnosed to have chronic obstructive pulmonary disease with recurrent attacks of dyspnoea and cough for the last 20 years. He reported swelling of the legs for the last 2 months. On examination, there were signs of chronic systemic venous congestion. Cardiac apex was found in the left 5^th^ intercostal space, 1cm lateral to mid-clavicular line. A left parasternal heave was noted with a palpable pulmonary component of second heart sound. On auscultation, the first heart sound was normal. The pulmonary component of second heart sound was accentuated. Indirect laryngoscopy revealed a paralysed left vocal fold. Chest X-ray showed findings suggestive of chronic obstructive pulmonary disease (COPD) with chronic cor pulmonale. Echocardiography showed a dilated pulmonary artery with right ventricular hypertrophy. The calculated pulmonary artery pressure was 27mm of Hg.

## DISCUSSION

The causes of recurrent laryngeal nerve paralysis in an ENT setting have been classified as non-surgical paralysis, surgical paralysis (thyroid/oesophageal operations and intubation); or a combination of the two^1^.

In 1897, Ortner described a series of 3 cases of mitral stenosis suffering from hoarseness of voice because of left recurrent laryngeal nerve palsy. He deduced the cause to be compression of the left recurrent laryngeal nerve by an enlarged left atrium^2^. Since then various authors have recorded their experiences of recurrent laryngeal nerve involvement in various cardiac disorders such as Eisenmenger complex^3^, left ventricular failure^4^, atrial septal defect^5^, patent ductus arteriosus (PDA)^6^,^7^, primary pulmonary hypertension^8^, ^9^, ^10^ recurrent pulmonary artery embolism^11^, mitral regurgitation^12^, atrial myxoma^13^, left ventricular aneurysm^14^, cor pulmonale^15^ and various types of aortic aneurysms^16^, ^17^, ^18^, ^19^, ^20^, ^21^.

A number of authors have questioned the explanation offered by Ortner to the cause of vocal fold palsy. The occurrence of neural compression between a dilated pulmonary artery and the aorta or aortic ligament was suggested based on X-ray and autopsy findings^6^. As more cases of hoarseness of voice with cardiac disorders were reported, more theories to its cause were described such as lymphadenitis and scarring in the aortic window causing nerve fixation, pressure from the left bronchus, right ventricular hypertrophy, pulmonary artery atherosclerosis, anatomical position of the ligamentum arteriosum^5^ or a dilated pulmonary artery^8^. It has been hypothesised by some that patients with arteriosclerotic heart diseases suddenly suffered left recurrent laryngeal nerve paralysis because rapid onset of left ventricular failure produced sudden pulmonary hypertension with acute dilatation of the pulmonary vessels. This phenomenon has been termed as dynamic dilatation. Hoarseness associated with acute cardiac exacerbation has been reported in patients with long-standing PDA, thus corroborating the above proposition^7^.

Since slow or partial injury to the nerve may not always result in hoarseness, routine examination of the vocal fold in all cases of heart disease has been advocated. If palsy of the left vocal fold is visualised then raised pulmonary artery pressure may be safely deduced^6^.

Owing to the rarity of vocal fold paralysis in heart disease, the importance of a constantly dilated pulmonary artery under tension has been stressed^22^,^23^.

## CONCLUSION

Ortner's syndrome also known as cardiovocal syndrome is a rare condition which may be secondary to many cardiopulmonary disorders. Pulmonary hypertension or some cause leading to dilatation and increased tension of the pulmonary artery, whether temporary or 'dynamic' may be responsible for vocal fold palsy. Nerve compression between the aorta and tense pulmonary artery is a constant factor in all cases. It would therefore be pertinent to look beyond the larynx for a cause of vocal cord palsy in patients presenting with hoarseness of voice and also routinely perform indirect laryngoscopy in all cases of heart disease.
